# Supplementary material for: The influence of environmental factors on the detection and quantification of SARS-CoV-2 variants in dormitory wastewater at a primarily undergraduate institution
Source: Microbiol Spectr. 2025 Jan 10;13(2):e02003-24. doi: 10.1128/spectrum.02003-24 (PMC11792549; doi:10.1128/spectrum.02003-24)
Supplement: Supplemental figures — Fig. S1 to S4. [file spectrum.02003-24-s0002.pdf]

SUPPLEMENTARY FIGURES

Title: The influence of environmental factors on the detection and quantification of SARS-CoV-2 Variants in Dormitory Wastewater at a Primarily Undergraduate Institution

Authors: Chequita Brooks[1,3], Sebrina Brooks[2], Josie Beasley[1], Jenna Valley[1], Michael Opata[1], Ece Karatan[1], Rachel Bleich[1#]

Author Affiliations:

1. Department of Biology, Appalachian State University, Boone, NC 28608
2. Department of Biology, University of North Carolina at Wilmington, Wilmington, NC 28403
3. Louisiana Universities Marine Consortium, Chauvin, LA 70344

Corresponding author:

Rachel Bleich, Appalachian State University, bleichrm@appstate.edu

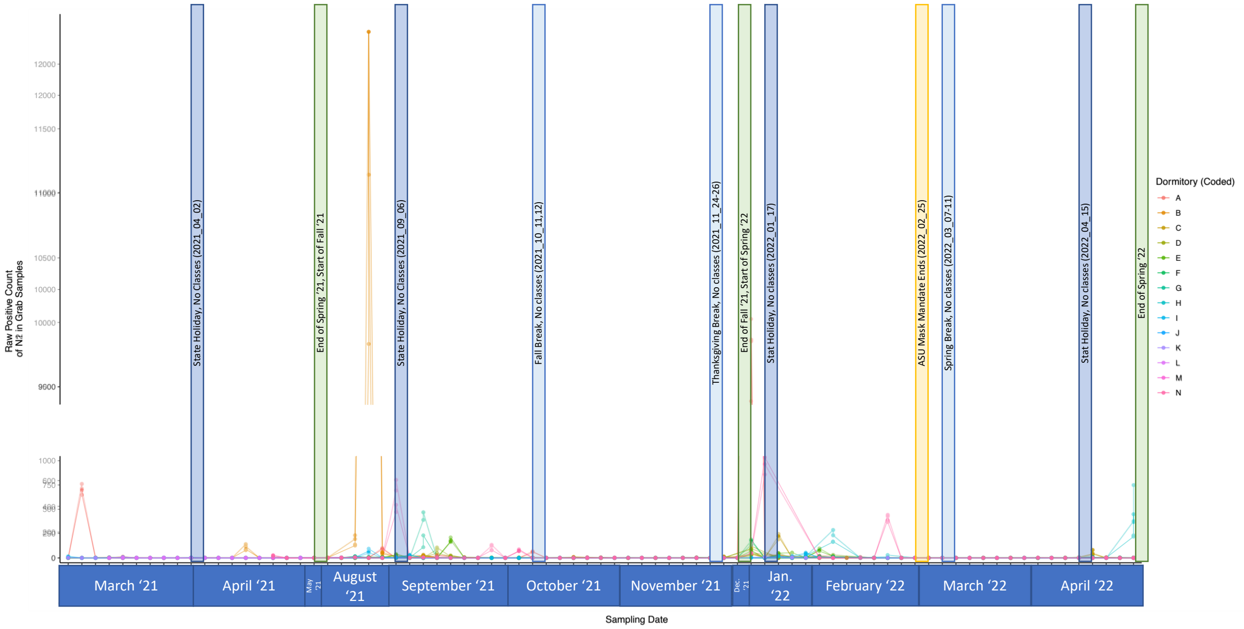

Figure S1. Raw positive count of N1and N2 (graphs overlaid) with all non-class days indicated.

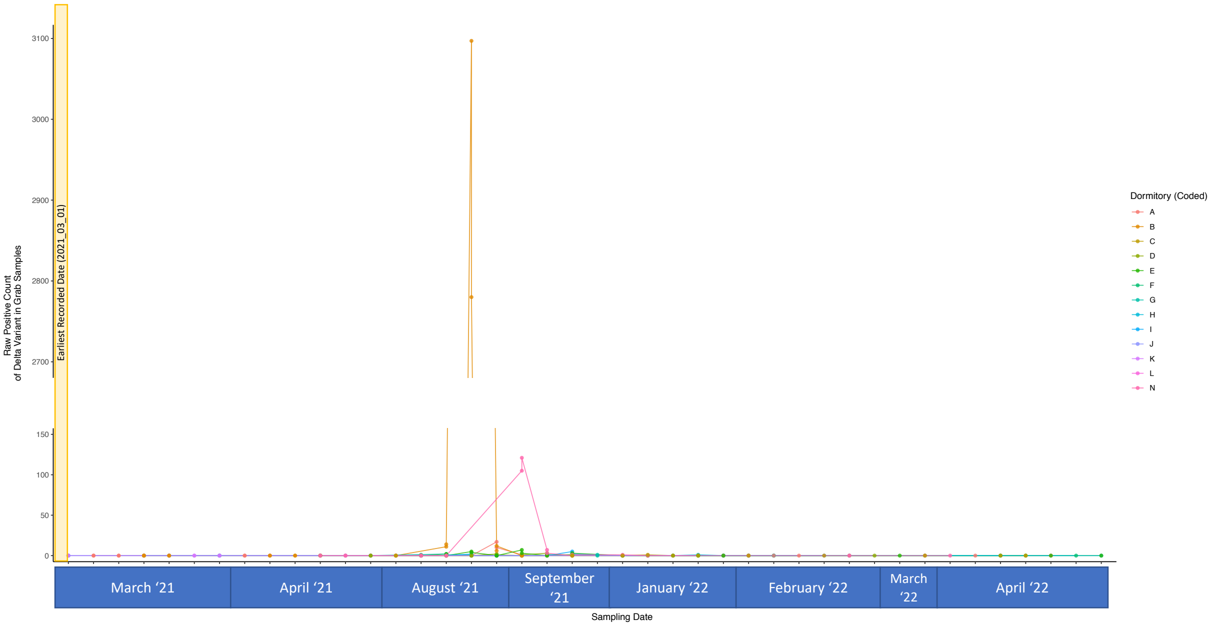

Figure S2. Raw positive count of the delta variant with the first known detection globally indicated.

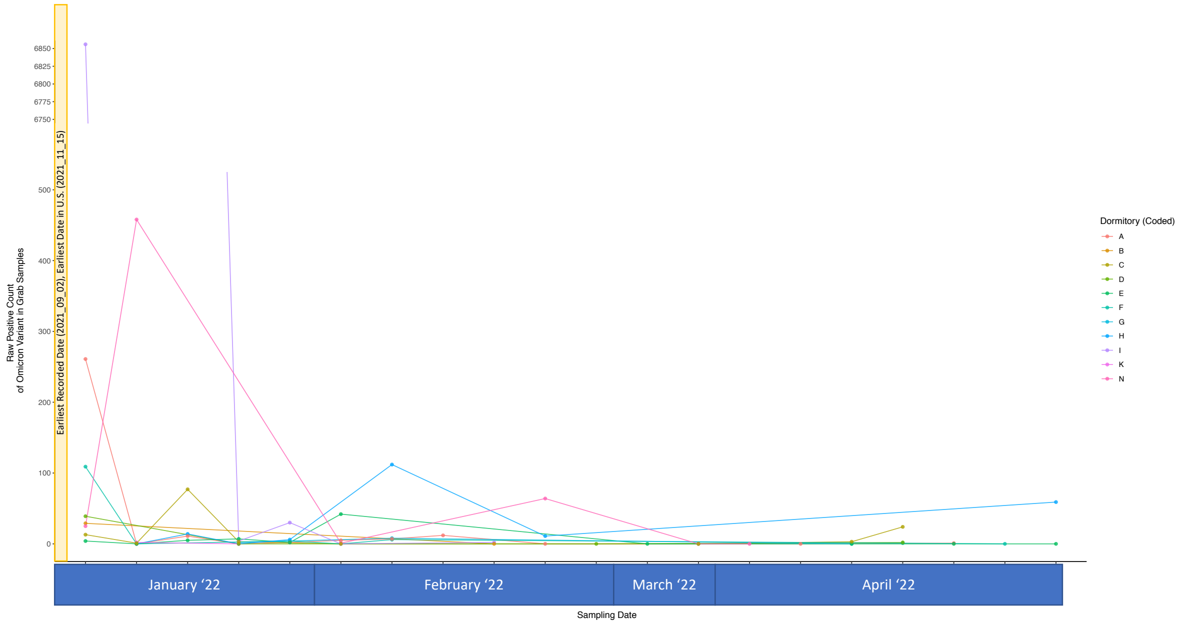

Figure S3. Raw positive count of the omicron variant with the first known detection globally indicated.

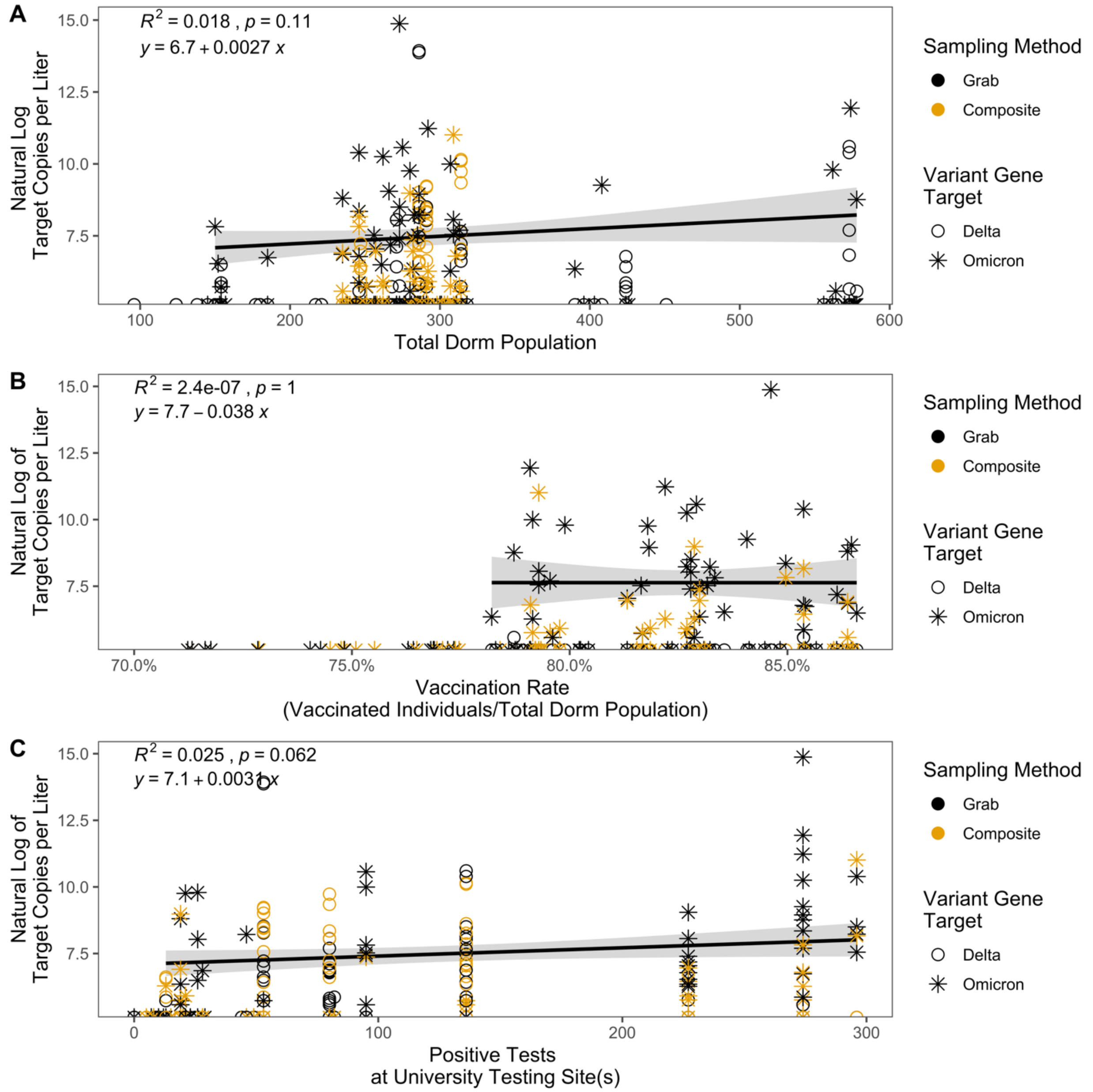

Figure S4. Correlation between the natural log of the target copies per liter for the variants of concern delta (open circle) and omicron (star) and sampling method (grab = black, composite = yellow) by A) total dormitory population, B) vaccination rate, and C) cumulative positive tests at university testing site(s).
